# Epidemiology of SARS-CoV2 in Qatar’s primary care population aged 10 years and above

**DOI:** 10.1186/s12879-021-06251-z

**Published:** 2021-07-05

**Authors:** Mohamed Ahmed Syed, Ahmed Sameer Al Nuaimi, Hamda Abdulla A/Qotba, Gheyath K. Nasrallah, Asmaa A. Althani, Hadi M. Yassine, Abduljaleel Abdullatif Zainel, Hanan Khudadad, Tamara Marji, Shajitha Thekke Veettil, Hadeel T. Al-Jighefee, Salma Younes, Farah Shurrab, Duaa W. Al-Sadeq, Al Anoud Saleh AlFehaidi, Ameena Ibrahim Yfakhroo, Meshal Abdulla AlMesaifri, Hanan Al Mujalli, Samya Ahmad Al Abdulla, Mohamed Ghaith Al Kuwari, Faruk Mohammed Azad, Badria Ali Mohamed Al Malki, Mariam Ali Abdulmalik

**Affiliations:** 1 Department of Clinical Research, Primary Health Care Corporation, P.O. Box 26555, Doha, Qatar; 2 Biomedical Research Center, Qatar University, P.O. Box 2713, Doha, Qatar; 3 Department of Biomedical Science, College of Health Sciences, Member of QU Health, Qatar University, P.O. Box 2713, Doha, Qatar; 4 Directorate of Clinical Affairs, Primary Health Care Corporation, P.O. Box 26555, Doha, Qatar; 5 Directorate of Clinical Operations, Primary Health Care Corporation, P.O. Box 26555, Doha, Qatar; 6 Directorate of Strategy Planning and Health Intelligence, Primary Health Care Corporation, P.O. Box 26555, Doha, Qatar; 7 Department of Health Affairs, Ministry of Public Health, P.O. Box 42, Doha, Qatar

**Keywords:** COVID-19, SARS-CoV2, Epidemiology, Primary care

## Abstract

**Background:**

There is an urgent need to elucidate the epidemiology of the Severe Acute Respiratory Syndrome Coronavirus 2 (SARS-CoV2) and characterize its potential impact. Investing in characterising the SARS-CoV2 will help plan and improve the response to the pandemic. Furthermore, it will help identify the most efficient ways of managing the pandemic, avoiding public health policies and interventions that may be unduly restrictive of normal activity or unnecessarily costly. This paper describes the design and reports findings of a population based epidemiological study undertaken to characterise SARS-CoV2 in Qatar using limited resources in a timely manner.

**Methods:**

Asymptomatic individuals ≥10 years registered with Qatar’s publicly funded primary health provider were eligible. A stratified random sampling technique was utilized to identify the study sample. Participants were invited to an appointment where they completed a questionnaire and provided samples for polymerase chain reaction and Immunoglobulin M and G immunoassay tests. Data collected were analyzed to calculate point and period prevalence by sociodemographic, lifestyle and clinical characteristics.

**Results:**

Of 18,918 individuals invited for the study, 2084 participated (response rate 10.8%). The overall point prevalence and period prevalence were estimated to be 1.6% (95% CI 1.1–2.2) and 14.6% (95% CI 13.1–16.2) respectively. Period prevalence of SARS-CoV2 infection was not considerably different across age groups (9.7–19.8%). It was higher in males compared to females (16.2 and 12.7% respectively). A significant variation was observed by nationality (7.1 to 22.2%) and municipalities (6.9–35.3%).

**Conclusions:**

The study provides an example of a methodologically robust approach that can be undertaken in a timely manner with limited resources. It reports much-needed epidemiological data about the spread of SARS-CoV2. Given the low prevalence rates, majority of the population in Qatar remains susceptible. Enhanced surveillance must continue to be in place, particularly due to the large number of asymptomatic cases observed. Robust contact tracing and social distancing measures are key to prevent future outbreaks.

**Supplementary Information:**

The online version contains supplementary material available at 10.1186/s12879-021-06251-z.

## Background

The first cases of Severe Acute Respiratory Syndrome Coronavirus 2 (SARS-CoV2), the causative agent for coronavirus disease 2019 (COVID-2019), were reported on 31st December 2019 in Wuhan (China) [1, 2]. As the disease continued to spread globally, the World Health Organisation (WHO) declared it a Public Health Emergency of International Concern (PHEIC) on the 30th January 2020 [3]. With 118,000 cases in 114 countries, and 4291 deaths, the WHO declared the SARS-CoV2 outbreak a pandemic on March 11, 2020 [4].

There is an urgent need to elucidate the epidemiology of the novel virus and characterize its potential impact [5]. Investing in characterising the SARS-CoV2 will help plan and improve the response to the pandemic. Furthermore, it will help identify the most efficient ways of managing the pandemic, avoiding public health policies and interventions that may be unduly restrictive of normal activity or unnecessarily costly. Majority of studies undertaken to date are based on a non-random sample, focus on specific population groups making it difficult to generalise findings or resource intensive.

The first COVID-19 cases in Qatar were reported on 29th of February 2020. Since the start of the epidemic in the country, as of 12th September, 691,031 individuals have been tested using a diagnostic polymerase chain reaction (PCR) test [6]. Of these 118,682 were found to be infected with SARS-CoV2. During the course of the pandemic, Qatar has had a high number of confirmed cases compared to any other country in the world relative to its population, and the second-highest total of confirmed cases in the Middle East after Saudi Arabia.

Despite the significant number of PCR tests conducted in Qatar, the precise number of infected individuals remains unclear due to factors related to the clinical presentation of coronavirus disease (COVID-19) and PCR testing. Serological tests have an advantage over PCR tests in this context. They can identify individuals who were previously infected with SARS-CoV2 even if they never underwent testing while acutely ill [7].

In July 2020, Primary Health Care Corporation in collaboration with Qatar University’s Biomedical Research Centre initiated a three-phase longitudinal epidemiological study to obtain prevalence estimates by sociodemographic, lifestyle and clinical characteristics. The study was designed to include a nationally representative, randomly selected sample requiring limited resources. This paper describes the study design and reports phase one findings.

## Methods

### Study settings

Qatar is a sovereign and independent state in the Middle East. It has eight municipalities which vary in area and population (Ad Dawhah, Al Rayyan, Al Daayen, Umm Salal, Al Khor, Al Shamal, Al Shahaniya and Al Wakrah). Qatar is known for its extensive development over recent years attracting expatriates from all over the world. It has a modern publicly funded healthcare system. This includes a universal publicly funded health care service delivered by the Primary Health Care Corporation (PHCC – responsible for primary health care) and Hamad Medical Corporation (HMC- responsible for secondary and tertiary care) for its registered population (Qataris and Expatriates). A small number of private clinics and hospitals also operate in the country. PHCC is the largest primary health care provider in Qatar. Majority of the country’s population is registered with PHCC. As of June 2020, a total of 1,461,759 individuals were registered across 27 health centres, all of which are accredited by Accreditation Canada.

### Study design and population

The study was designed to include three phases. A 3-month time interval was planned between each phase. Each phase was planned to be conducted within a three-week time duration. Asymptomatic individuals registered with a mobile number on PHCC electronic medical records and ≥ 10 years of age were eligible for inclusion. Individuals below 10 years were excluded for practical reasons. Individuals with difficulties related to mobility and communication, bleeding disorders and mental disabilities were excluded.

A stratified random sampling technique was utilized to identify the study sample. 16 strata were defined using age, gender nationality and their sub-categories. The sample size for each stratum was calculated using the total PHCC registered population within it. The required total sample size for the study was estimated to be 2102. Further details of stratification and sample size calculation are provided in the supplementary file.

### Participant recruitment

A full list of eligible participants (*N* = 1,063,243) was extracted from PHCC’s electronic medical records system with their health record number, name, age, gender, nationality and mobile phone number. Participants were randomly selected to create 5 sets of the required sample in anticipation of a non-response rate of 80%. Participants were sent Short Message Service (SMS) messages on their mobile phones inviting them to participate in the study. Recruitment rates were reviewed at the end of week 1 and week 2 during the 3-week study duration to facilitate targeted invitations by strata in the following week.

### Study locations and data collection

Participants were invited to an appointment at one of three PHCC health centres (Qatar University, Al Wakrah and Al Wajbah) identified for the study which operated in two shifts (8 am – 2 pm and 4 pm – 8 pm). In each of the health centres, a separate room with a direct entrance from outside was set up to see patients. The room was divided in 3 stations – Patient registration, patient interview and sample collection station. The rooms were set up as such to facilitate smooth patient flow while adhering to strict social distancing and infection control protocols.

On arrival for their appointment, participants were registered on the electronic medical records system at the registration station. Following registration, at the patient interview station, a member of the research team briefed the participant about the study and obtained written informed consent from adults or assent from minors. Participants were administered an electronic interview based questionnaire that included questions on sociodemographic factors (education level, employment status, number of rooms and individuals in household), lifestyle (physical activity, smoking status, fruit consumption, vegetable or salad consumption, fruit juice consumption), history of chronic conditions (supplementary file), Bacillus Calmette–Guérin (BGC) and influenza vaccine status, previous RT-PCR status, COVID-19 related symptoms and contact with suspected or confirmed cases. Participants' height and weight were also recorded. At the sample collection station, nasal and oropharyngeal swab and blood samples were collected. Prior training was provided to all data collection personnel. They were always supervised by a core research team member to ensure adherence to agreed common protocols.

### Laboratory procedures

Both nasal and oropharyngeal swab samples were collected from all participants. The swabs were labelled and transported from the study location to the referral laboratory for the state of Qatar’s at the end of each shift. RNA was extracted and isolated prior to amplification on a number of platforms. These included: ExiPrep 96 Viral DNA/RNA Kit (Bioneer Corporation, South Korea Cat Number K-4614); Chemagic STARS Viral DNA/RNA 300 Kit (Perkin Elmer, US, Cat Number CMG-1774); EZ1 Virus Mini Kit v2.0 (QIAGEN, US Cat Number 955134); Nucleic Acid Extraction Kit (Wuhan MGI Tech Co Ltd., China (Cat Number 1000021043).

Extracted nucleic acid underwent thermal cycling on ABI 7500 Thermal Cyclers (ThermoFischer, US). using a number of thermal mixes. These included: Taqpath Covid 19 RT PCR (ThermoFisher, US, A48102); AccuPower SARS-COV2 RT PCR (Bioneer Corporation, South Korea Cat Number SCV-2122); GeneFinder (OSANG Healthcare Co., Ltd. China, Cat Number IFMR-45. Additionally, 2 fully automated systems combining nucleic acid extraction and thermal cycling were operational. These included: Cobas SARS-CoV2 (Roche, Germany, Cat Number P/N:09175431190); GeneXpert SARS-CoV2 (Cepheid, US Cat Number XPRSARS-COV2–10). A total of 5 different RNA genes in different combinations as dictated by the commercial suppliers were amplified. Each assay was validated for cycle threshold (CT) value interpretation using the manufacturer’s instructions.

Blood samples were labelled and stored at the study location and transported every 24 h to Qatar University’s Biomedical Research Centre laboratory where plasma was separated by centrifugation. 150 μL of plasma was used for detection of anti-SARS-COV2 Immunoglobulin G (IgG) using the CL-900i Chemiluminescence Immunoassay System (Mindray Bio-Medical Electronics Co, Shenzen, China) according to the manufactures instructions. Immunoglobulins were directed against the spike protein (S subunit). The CL-series SARS-COV2 IgG assay is designed to have a precision of ≤10%. Precision was determined according National Committee for Clinical Laboratory Standards (NCCLS) Protocol EP15-A37 (as specified by the manufacturer). The analytical performance of CL-series SARS-CoV2 IgG immunoassay (Cat. No. SARS-CoV2 IgG121) was evaluated using previous RT-PCR test (as a gold standard) results stratified by the symptom category at the time of swab (supplementary file).

### Notification of laboratory results

Participants with negative RT-PCR test results were notified by SMS messages. Participants with positive RT-PCR test results were contacted by the Qatar’s Ministry of Public Health for further advice and care provided as required. All blood test results were notified by SMS.

### Data analysis

All data was collated at the end of phase one. It was subject to quality assurance. For the purposes of this study, point prevalence was defined as the number of active asymptomatic SARS-CoV2 infections (identified by RT-PCR) over the total sample size while period prevalence was defined as the total number of SARS-CoV2 infections [either identified by RT-PCR or serology (IgM and IgG)] over the total sample size. Initial analysis was undertaken to establish the overall point and period prevalence. Further analysis was undertaken to estimate prevalence by age, gender, nationality (supplementary file) and municipalities. Period prevalence was also estimated by sociodemographic [education level, employment status, household crowding index (HCI)], lifestyle [body mass index (BMI), physical activitysmoking status, fruit consumption, vegetable or salad consumption and fruit juice] and clinical characteristics (BGC and seasonal influenza vaccine status, history of chronic diseases COVID-19 related symptoms and previous contact with suspected or confirmed case).

The 95% confidence intervals (CIs) were calculated for proportions (prevalence rates). Chi-square test of independence was used to assess the statistical significance of associations between nominal or ordinal scale variables. To measure the strength of association between a dichotomous independent variable (a specific group compared to reference group) and a dichotomous outcome variable (having COVID-19) the prevalence ratio (PR) was used. The logarithm method was used in calculation of confidence intervals for the calculated PR. *P* value less than the 0.05 level of significance was considered statistically significant. All statistical analyses were done using survey commands in IBM SPSS Statistics for Windows (Version 23.0. Armonk, NY: IBM Corp.)..

### Ethical considerations

The study was reviewed and approved by Primary Health Care Corporation’s Independent Review Board (Reference number PHCCDCR202005047). Written informed assent was obtained from participants aged 10–18 years and written informed consent was obtained from participants aged 18 or over. Only MAS, ASAN and HAQ had access to the full study data. Overall, the study was conducted with integrity according to generally accepted ethical principles.

## Results

### Sample recruitment

Of 18,918 individuals invited for the study, 2044 participated (response rate 10.8%). The recruited sample was 97.2% complete compared to the initially planned study sample (Table 1). Overall, more Qataris were recruited by 6.4%) while expatriates were under recruited by 2.8%. Qatari female 18–49 years (*N* = − 27; − 25.5%), Qatari females ≥60 years (N = -8; − 34.8%) and expatriate females 18–49 years (*N* = − 98; − 21.5%) were the most under recruited. Qatari males 10–17 years (*N* = 24; 44.4%), Qatari females 10–17 years *N* = 18; 34.6%), and expatriates male ≥60 years (*N* = 10; 18.9%), were the most over recruited.

**Table 1:** Sample recruitment by strata

Nationality and Gender	Age group (years)	Target sample	Total participated	Deviation from target sample
		N	N	N (%)
Qatar				
Female	10–17	52	70	18 (34.6)
Male		54	78	24 (44.4)
Female	18–49	106	79	−27 (−25.5)
Male		100	93	−7 (− 7)
Female	40–59	55	72	17 (30.9)
Male		44	53	9 (20.5)
Female	≥ 60	23	15	−8 (−34.8)
Male		19	22	3 (15.8)
Total Qatar		**453**	**482**	**29 (6.4)**
Expatriate				
Female	10–17	98	91	−7 (−7.1)
Male		105	113	8 (7.6)
Female	18–49	456	358	−98 (−21.5)
Male		412	380	−32 (−7.8)
Female	40–59	206	232	26 (12.6)
Male		294	299	5 (1.7)
Female	≥ 60	25	26	1 (4)
Male		53	63	10 (18.9)
Total Expatriate		**1649**	**1562**	**−87 (−5.3)**
Grand Total		**2102**	**2044**	**−58 (−2.8)**

### Overall point and period prevalence

Based on the information collected in the questionnaire, it was found that 3.6% (*N* = 74) study participants had previously tested positive by RT-PCR (Table 2). Of the 2044 participants, 1.6% were found to have an active SARS-CoV2 infection by RT-PCR and 13.3% (*N* = 272) were found to have had a previous SARS-CoV2 infection by serology. Based on these findings, in Qatar, the overall point prevalence was estimated to be 1.6% (95% CI 1.1–2.2) and period prevalence was estimated to be 14.6% (95% CI 13.1–16.2) (Table 2).

**Table 2:** Prevalence of SARS-CoV2 infection (*N* = 2044)

Previous RT-PCR	Serology (IgM/IgG)	Point Prevalence	Period Prevalence
% [N] (95% CI)	% [N] (95% CI)	% [N] (95% CI)	% [N] (95% CI)
3.6 [74] (2.9 to 4.5)	13.3 [272] (11.9 to 14.8)	1.6 [32] (1.1 to 2.2)	14.6 [298] (13.1 to 16.2)

### Point prevalence of SARS-CoV2 by age, gender, nationality and municipality

Point prevalence was found to be almost similar across all ages (range 1.4–1.9%) except in ≥60 (0%) (Table 3). Furthermore, point prevalence between females (1.5%; 95% CI 0.8–2.4%) and males (1.6%; 95% CI 1–2.5%) was found to be almost similar. Active SARS-CoV2 infection was found to be highest in North American (3.1%; 95% CI 0.3–13.7%) and Southern Asian nationalities (3%; 95% CI 1.7–4.8%). Point prevalence was lowest in Qatari and Northern African nationalities (0.6 and 0.7% respectively). In terms of geographic region. Al Khor municipality had the highest point prevalence (6.3%; 95% CI 1.8–15.7%) compared to all other municipalities in Qatar.

**Table 3:** Point prevalence of SARS-CoV2 by age, gender, nationality and municipality

	Total tested	Positive results	Point prevalence
	N	N	% (95% CI)
Age group (years)			
10–17	352	5	1.4 (0.5–3.1)
18–39	910	17	1.9 (1.1–2.9)
40–59	656	10	1.5 (0.8–2.7)
≥ 60	126	0	N/A
Gender			
Female	943	14	1.5 (0.9–2.4)
Male	1101	18	1.6 (1–2.5)
Nationality ^α^			
Qatar	482	3	0.6 (0.2–1.7)
Northern Africa	410	3	0.7 (0.2–1.9)
South-eastern Asia	137	2	1.5 (0.3–4.6)
Southern Asia	468	14	3 (1.7–4.8)
Western Asia	375	7	1.9 (0.8–3.6)
Northern America	32	1	3.1 (0.3–13.7)
Europe	87	1	1.1 (0.1–5.2)
Others	53	1	1.9 (0.2–8.5)
Municipality ^β^			
Ad-Dawhah Municipality	746	10	1.3 (0.7–2.4)
Al Rayyan Municipality	655	14	2.1 (1.2–3.5)
Al Daayen Municipality	116	0	N/A
Umm Salal Municipality	130	2	1.5 (0.3–4.8)
Al Khor Municipality	48	3	6.3 (1.8–15.7)
Al Shamal Municipality	5	0	N/A
Al-Shahaniya Municipality	17	0	N/A
Al Wakrah Municipality	221	1	0.5 (0–2.1)

^α^ Full list of nationality categories is provided in the supplementary file

^β^ Municipality information was not available for 130 participants (6.2% of the study sample)

### Period prevalence of SARS-CoV2 by age, gender, nationality and municipality

Period prevalence of SARS-CoV2 infection was not considerably different across age groups (Table 4). It ranged from 14.4–19.8% in participants aged 18 and above. It was lowest in 10–17 age group (9.7%; 95% 6.9–13.1%). SARS-CoV2 infection rate in males was higher than that of females (16. 3 and 12.7% respectively). A significant variation of infection rates was observed by nationality. It ranged from 5.7 to 22.2% with lowest rates in European (5.7%; 95% CI 2.2–12.1%) and Qatari nationalities (7.1%; 5–9.6%). Highest rates were found in Northern African nationalities (19.3%: 95% CI 15.7–23.3%) and Southern Asian (22.2%; 95% CI 18.6–26.2%). By geographic region, Al Shahaniya (35.3%; 95% 16.3–58.9%) and Al Shamal (40%; 9.4–79.1%) municipalities had the highest prevalence rates while Al Daayen municipality had the lowest prevalence rates (6.9%; 95% CI 3.3–12.6%).

**Table 4:** Period prevalence of SARS-CoV2 by age, gender, nationality and municipality

	Total Tested	Positive Results	Period Prevalence
	N	N	% (95% CI)
Age group (years)			
10–17	352	34	9.7 (6.9–13.1)
18–39	910	131	14.4 (12.2–16.8)
40–59	656	108	16.5 (13.8–19.4)
≥ 60	126	25	19.8 (13.6–27.4)
Gender			
Female	943	120	12.7 (10.7–15)
Male	1101	178	16.2 (14.1–18.4)
Nationality ^**α**^			
Qatar	482	34	7.1 (5–9.6)
Northern Africa	410	79	19.3 (15.7–23.3)
South-eastern Asia	137	19	13.9 (8.9–20.4)
Southern Asia	468	104	22.2 (18.6–26.2)
Western Asia	375	47	12.5 (9.5–16.2)
Northern America	32	5	15.6 (6.2–30.9)
Europe	87	5	5.7 (2.2–12.1)
Others	53	5	9.4 (3.7–19.4)
Municipality ^**β**^			
Ad-Dawhah Municipality	746	114	15.3 (12.8–18)
Al RayyanMunicipality	655	86	13.1 (10.7–15.9)
Al Daayen Municipality	116	8	6.9 (3.3–12.6)
Umm Salal Municipality	130	18	13.8 (8.7–20.6)
Al Khor Municipality	48	10	20.8 (11.2–33.8)
Al Shamal Municipality	5	2	40 (9.4–79.1)
Al-Shahaniya Municipality	17	6	35.3 (16.3–58.9)
Al Wakrah Municipality	221	29	13.1 (9.2–18)

^α^ Full list of nationality categories is provided in the supplementary file

^β^ Municipality information was not available for 130 participants (6.2% of the study sample)

### Period prevalence of SARS-CoV2 by sociodemographic, lifestyle and clinical characteristics

Period prevalence of SARS-CoV2 infection did not differ by education levels except in participants with a technical or trade qualification, who were found to have a higher prevalence (20.5%) (Table 5). However, a notable difference in prevalence was seen by employment status. Participants who were retired (6.7%) or students (9.1%) had the lowest prevalence. Similarly, participants with a higher household crowding index had a higher prevalence of SARS-CoV2 infection (≤ 1 = 13.4%; 1.1–2 = 14.5% and > 2 = 20.6%).

**Table 5:** Period prevalence of SARS-CoV2 by sociodemographic, lifestyle and clinical characteristics

	Total tested	Positive results	Period prevalence
	N	N	% (95% CI)
Highest education level			
Never attended school	19	3	15.8 (4.7–36.4)
School	762	102	13.4 (11.1–15.9)
Technical or trade qualification	78	16	20.5 (12.7–30.4)
University	1174	176	15 (13–17.1)
Employment status			
Employed	1239	201	16.2 (14.2–18.4)
Unemployed	291	47	16.2 (12.3–20.7)
Student	417	38	9.1 (6.6–12.2)
Retired	45	3	6.7 (1.9–16.7)
Household crowding index ^¥^			
≤ 1	867	116	13.4 (11.2–15.8)
1.1–2	958	139	14.5 (12.4–16.8)
> 2	204	42	20.6 (15.5–26.5)
BMI (kg/m^2^)			
Healthy (18.5–24.9)	620	75	12.1 (9.7–14.8)
Underweight (< 18.5)	28	1	3.6 (0.4–15.5)
Overweight (25 to 29.9)	742	122	16.4 (13.9–19.2)
Obese (≥ 30.0)	633	98	15.5 (12.8–18.5)
Smoking status			
Non-smoker	1597	237	14.8 (13.2–16.6)
Ex- smoker	137	24	17.5 (11.9–24.5)
Current smoker	285	35	12.3 (8.9–16.5)
Current smoking index ^◊^			
Lowest trecile	94	19	20.2 (13.1–29.2)
Intermediary trecile	87	7	8 (3.7–15.1)
Third trecile	88	9	10.2 (5.2–17.8)
Physical activity			
0 min/week	928	129	13.9 (11.8–16.2)
1–150 min/week	431	72	16.7 (13.4–20.4)
150–300 min/week	386	54	14 (10.8–17.7)
> 300 min/week	285	42	14.7 (11–19.2)
Fruit consumption			
Less than once a week	137	12	8.8 (4.9–14.4)
2–7 times a week	705	95	13.5 (11.1–16.1)
Once or more than once a day	1187	190	16 (14–18.2)
Vegetable or salad consumption			
Less than once a week	105	6	5.7 (2.4–11.4)
2–7 times a week	632	97	15.3 (12.7–18.3)
Once or more than once a day	1292	194	15 (13.1–17)
Fruit juice consumption			
Less than once a week	669	106	15.8 (13.2–18.8)
2–7 times a week	828	123	14.9 (12.6–17.4)
Once or more than once a day	426	55	12.9 (10–16.3)
BGC vaccination			
No	121	18	14.9 (9.4–22)
Yes	1838	264	14.4 (12.8–16)
Seasonal influenza vaccination in previous 12 months			
No	1579	228	14.4 (12.8–16.2)
Yes	411	65	15.8 (12.5–19.6)
History of chronic conditions			
0	1485	203	13.7 (12–15.5)
1	367	61	16.6 (13.1–20.7)
2	132	21	15.9 (10.4–22.9)
3 or more	49	12	24.5 (14.1–37.8)
COVID-19 symptoms since start of pandemic ^∞^			
Asymptomatic	1684	195	11.6 (10.1–13.2)
Paucisymptomatic	226	52	23 (17.9–28.8)
Symptomatic	123	50	40.7 (32.3–49.5)
Previous close contact with an individual suspected or confirmed COVID-19			
No	1849	235	12.7 (11.3–14.3)
Yes	160	59	36.9 (29.7–44.5)

^**¥**^ Defined as the total number of co-residents per household, excluding the newborn infant, divided by the total number of rooms, excluding the kitchen and bathrooms.^**◊**^ Defined as average count of cigarettes smoked / day (coded as 1 = < 2, 2 = 2–10, 3 = 11–20, 4 = 21–30, 5 = 31–40, 6 = 51–60) X duration of cigarette smoking in years

^**∞**^ Asymptomatic defined as no symptoms, paucisymptomatic defined as 1–2 symptoms without anosmia or ageusia) and symptomatic defined as anosmia or ageusia, or at least three symptoms among fever; chills; severe tiredness; sore throat; cough; shortness of breath; headache; or nausea, vomiting, or diarrhoea

Slightly higher prevalence was seen in participants who were either overweight (16.4%) or obese (15.5%) compared to healthy (12.1%) participants. Ex-smokers showed slightly higher prevalence (17.5%) compared to, current (12.3%) and non-smokers (14.8%). In current smokers, those who smoked more had a lower prevalence (8–10.2%) compared to those who smoked least (20.2%). No significant differences were seen related to physical activity or fruit juice consumption. Consuming fruits or vegetable or salads more than once a week found to have lower prevalence rates 8.8% (95% CI 4.9–14.4%) and 5.7 (95% CI 2.4–11.4) respectively.

A history of a BCG or influenza vaccination in previous 12 months did not show a difference in prevalence amongst participants (BCG Yes- 14.4% and No − 14.9; Influenza Yes- 15.8 and No – 14.4). However, a history of 3 or more chronic conditions showed a higher prevalence (24.5%) compared to having two or less chronic conditions (13.7–16.6%). The prevalence was higher in participants who had one or more COVID-19 symptoms since the start of the pandemic (40.7%) compared to those who were asymptomatic (11.6%). Similarly, the prevalence was higher in those who had close contact with an individual suspect or confirmed of COVID-19 (36.9%) compared with those who did not (12.7%).

## Discussion

This study provides an example of a robust nationwide study undertaken using limited resource to accurately estimate the prevalence of SARS-CoV2 in Qatar. It estimated the overall point and period prevalence of SARS-CoV2 as 1.6 and 14.6% respectively. The point prevalence results suggest asymptomatic SARS-CoV2 infection in the population are at the lower end of the range (1.2 - 12.9%) reported by a systematic review [8]. More importantly, despite the large number of RT-PCR tests undertaken in the country, the study found a considerable difference in previous RT-PCR positive test results (3.6%) and positive serology test results (13.3%). This highlights the limitations of RT-PCR as a screening test unless it is undertaken using a robust sampling approach.

Given the study was designed to represent Qatar’s population, it was able to demonstrate notable differences in period prevalence of SARS-CoV2 infection by age, gender, nationality and municipality. The prevalence rates patterns by age were similar to that reported by ENE-COVID, a Spanish population-based seroprevalence study [9]. Individuals aged 10–17 years had the lowest period prevalence (9.7%) while it was higher but similar across other age groups (10.6–11.9%). These findings are in line with previously reported studies [10, 11]. The prevalence was twice or more across all age groups compared to the ENE-COVID study [12]. Despite a higher prevalence, Qatar experienced fewer deaths per million (66 per million) compared to Spain (615 per million) [12].

While the study did not include a large sample size in terms of participant numbers compared to other studies, it is comparable to the largest population-based study undertaken to date (ENE-COVID study) in terms of participants per million population (1922 and 1299 respectively). Collection of nasal and oropharyngeal swab samples together with blood samples was an added benefit of the study. It allowed estimation of point prevalence in addition to the period prevalence of SARS-CoV2 infection in the population. At the same time exclusion of symptomatic individuals from the study may have underestimated point prevalence of SARS-CoV2 infections in the population.

In current smokers, prevalence was noted to decrease the more individuals smoked. Farsalinos et al. [13], in a systematic review, reported a low prevalence of current smoking was observed among patients with COVID-19. The review suggests the findings could potentially be due to research that suggest nicotine inhibits the production of pro-inflammatory cytokines and can potentially prevent acute lung injury. Further research is needed to better understand the effect of smoking in relation to SARS-CoV2 infection.

Having a history of symptoms showed a higher prevalence of SARS-CoV2 infection in this study. These findings are supported by a review with 24,410 adults with laboratory confirmed SARS-CoV2 infection from 9 countries where the authors reported that fever and a new persistent cough are the most prevalent symptoms of COVID-19 worldwide (78 and 57% respectively) [14]. Studies report between 40.8–57.7% prevalence of comorbidities in COVID-19 patients [15, 16]. This study found that individuals with more than three chronic conditions have a higher prevalence of SARS-CoV2 infection. It is suggested that there are several factors which might lead to increased susceptibility to SARS-CoV2 infections in individuals with chronic diseases. These include enhanced expression of the angiotensin-converting enzyme-2 in specific organs, cytokine storm, and drug interactions [17]. The relationship between the number of chronic conditions and SARS-CoV2 needs to be researched further.

The random selection of participants from PHCC registered population using electronic medical records is a key strength of the study. However, the invitation approach used resulted in the exclusion of individuals who did not register with PHCC using a mobile number and also restricted inclusion of those who are not technology savvy. Similarly, having only three study locations may have deterred individuals from participation if they were unable to travel to them. While the invitation approach and limited study locations are weaknesses of the study, they enabled its quick set up and execution without the need for huge resources. Questions on sociodemographic, lifestyle and clinical characteristics were self-reported which has limitations.

The SARS-CoV2 immunoassay used in the study had a specificity and sensitivity of 97.1% and 90.4% respectively. These are lower compared to other immunoassays with higher specificities (100%) and sensitivities (95%) [18]. This may have resulted in an underestimation of period prevalence.

## Conclusion

In conclusion, the study provides an example of a methodologically robust approach that can be undertaken in a timely manner with limited resources. It provides much-needed epidemiological data about SARS-CoV2 spread which are necessary to inform public health policies and interventions in Qatar as well as globally. Given the low prevalence rates, majority of the population in Qatar remains susceptible. Enhanced surveillance must continue to be in place, particularly due to the large number of asymptomatic cases observed. Robust contact tracing and social distancing measures are key to prevent future outbreaks.

## Supplementary Information

**Additional file 1: Table S1.**. Study strata. **Table S2.** Sample size determination and response rate by strata. **Table S3.** Validity parameters for IgG serology. **Table S4.** Nationality categories. **Table S5.** Point prevalence ratios of SARS-CoV2 by age, gender, nationality and municipality. **Table S6.** Period prevalence ratios of SARS-CoV2 by age, gender, nationality and municipality. **Table S7.** Period prevalence ratios of SARS-CoV2 by sociodemographic, lifestyle and clinical characteristics.

## Data Availability

The datasets used and/or analysed during the current study are available from the corresponding author on reasonable request.

## Abbreviations

SARS-CoV2: Severe Acute Respiratory Syndrome Coronavirus 2
COVID-19: Coronavirus Disease 2019
WHO: World Health Organisation
PHEIC: Public Health Emergency of International Concern
PCR: Polymerase chain reaction
PHCC: Primary Health Care Corporation
HMC: Hamad Medical Corporation
SMS: Short Message Service
BGC: Bacillus Calmette–Guérin
BMI: Body mass index
HCI: Household crowding index
