# Supplementary material for: Epidemiology of SARS-CoV2 in Qatar’s primary care population aged 10 years and above
Source: BMC Infect Dis. 2021 Jul 5;21:645. doi: 10.1186/s12879-021-06251-z (PMC8256211; doi:10.1186/s12879-021-06251-z)
Supplement: Supplementary file 1 — Additional file 1: Table S1.. Study strata. Table S2. Sample size determination and response rate by strata. Table S3. Validity parameters for IgG serology. Table S4. Nationality categories. Table S5. Point prevalence ratios of SARS-CoV2 by age, gender, nationality and municipality. Table S6. Period prevalence ratios of SARS-CoV2 by age, gender, nationality and municipality. Table S7. Period prevalence ratios of SARS-CoV2 by sociodemographic, lifestyle and clinical characteristics. [file 12879_2021_6251_MOESM1_ESM.docx]

**SUPPLEMENTARY APPENDIX**

This appendix has been provided by the authors to give readers additional information about their work.

Supplement to: Epidemiology of SARS-CoV2 in Qatar’s primary care population aged 10 years and above

**Contents**

[Study design 3](#_Toc49337032)

[Sample size determination 4](#_Toc49337033)

[List of chronic conditions 5](#_Toc49337034)

[Validity of serology test 6](#_Toc49337035)

[Nationality categories 7](#_Toc49337036)

[Prevalence ratios by age gender, nationality and municipality 9](#_Toc49337037)

[Prevalence ratio by sociodemographic, lifestyle and clinical characteristics 11](#_Toc49337038)

## Study design

Participants for the study were identified using PHCC’s electronic medical records. A full list of eligible participants (N= 1,063,243) was extracted with their health record number, name, age, gender, nationality and mobile phone number. A stratified random sampling technique was utilized to identify the study sample. 16 strata were defined using age, gender nationality and their sub-categories (Supplementary Table 1). The sample size for each stratum was calculated using the total PHCC registered population within it. The required total sample size for the study was estimated to be 2102.

Table S1: Study strata

| **Strata** | **Categories** |
| --- | --- |
| Age group (years) | 10-17 |
|  | 18-39 |
|  | 40-59 |
|  | ≥ 60 |
| Gender | Female |
|  | Male |
| Nationality | Qatar |
|  | Expatriate |

## Sample size determination

The sampling weights reference categories the proportion of the specific strata from the total PHCC population surveyed.

Table S2: Sample size determination and response rate by strata

| **Strata** | **Nationality** | **Gender** | **Age group** | **Sampling frame** | **Sampling fraction** | **Target sample** | **Sample invited** | **Total participated** | **Response rate** |
| --- | --- | --- | --- | --- | --- | --- | --- | --- | --- |
| 1 | Expatriate | Female | 10-17 years | 49397 | 0.046459 | 98 | 490 | 91 | 18.6 |
| 2 | Expatriate | Female | 18-39 years | 230986 | 0.217247 | 456 | 2280 | 358 | 15.7 |
| 3 | Expatriate | Female | 40-59 years | 104261 | 0.098059 | 206 | 1030 | 232 | 22.5 |
| 4 | Expatriate | Female | 60+ years | 12522 | 0.011777 | 25 | 125 | 26 | 20.8 |
| 5 | Expatriate | Male | 10-17 years | 53129 | 0.049969 | 105 | 525 | 113 | 21.5 |
| 6 | Expatriate | Male | 18-39 years | 208779 | 0.196361 | 412 | 2060 | 380 | 18.4 |
| 7 | Expatriate | Male | 40-59 years | 148697 | 0.139852 | 294 | 1470 | 299 | 20.3 |
| 8 | Expatriate | Male | 60+ years | 26713 | 0.025124 | 53 | 265 | 63 | 23.8 |
| 9 | Qatar | Female | 10-17 years | 26270 | 0.024707 | 52 | 260 | 70 | 26.9 |
| 10 | Qatar | Female | 18-39 years | 53481 | 0.050300 | 106 | 530 | 79 | 14.9 |
| 11 | Qatar | Female | 40-59 years | 27706 | 0.026058 | 55 | 275 | 72 | 26.2 |
| 12 | Qatar | Female | 60+ years | 11394 | 0.010716 | 23 | 115 | 15 | 13 |
| 13 | Qatar | Male | 10-17 years | 27427 | 0.025796 | 54 | 270 | 78 | 28.9 |
| 14 | Qatar | Male | 18-39 years | 50449 | 0.047448 | 100 | 500 | 93 | 18.6 |
| 15 | Qatar | Male | 40-59 years | 22521 | 0.021181 | 44 | 220 | 53 | 24.1 |
| 16 | Qatar | Male | 60+ years | 9511 | 0.008945 | 19 | 95 | 22 | 23.2 |
|  | **Total** |  |  | **1063243** | **1.00000** | **2102** | **10510** | **2044** | **19.4** |

## List of chronic conditions

Data on the below list of chronic conditions was collected in the questionnaire.

1. Cancer
2. Hypertension
3. Other cardiovascular conditions (Coronary artery disease/Congestive heart failure)
4. Pulmonary conditions (Asthma / Chronic obstructive pulmonary disease / Obstructive sleep apnea)
5. Renal conditions (Chronic kidney disease / End-staged kidney disease)
6. Liver conditions (Cirrhosis / Chronic liver disease / Hepatitis B / Hepatitis C)
7. Diabetes Mellitus
8. History of solid organ transplant
9. Thyroid disease (Hypo / hyper-thyroidism, ‎Goiter)
10. Dyslipidemia
11. Rheumatic/SLE/connective tissue disease

## Validity of serology test

Table S3: Validity parameters for IgG serology

| **IgG serology test** | **Previous rt-PCR test** | | | | |
| --- | --- | --- | --- | --- | --- |
|  | **Negative** | **Positive** | **Total** | **Sensitivity** | **Specificity** |
| **Asymptomatic ^∞^** |  |  |  | 81.0 | 94.7 |
| Negative | 142 | 4 | 146 |  |  |
| Positive | 6 | 17 | 23 |  |  |
| Total | 148 | 21 | 169 |  |  |
|  |  |  |  |  |  |
| **Pauci-symptomatic ^∞^** |  |  |  | 85.7 | 81.0 |
| Negative | 17 | 2 | 19 |  |  |
| Positive | 4 | 12 | 16 |  |  |
| Total | 21 | 14 | 35 |  |  |
|  |  |  |  |  |  |
| **Symptomatic ^∞^** |  |  |  | 92.3 | 100.0 |
| Negative | 9 | 3 | 12 |  |  |
| Positive | 0 | 36 | 36 |  |  |
| Total | 9 | 39 | 48 |  |  |
|  |  |  |  |  |  |
| **Overall** |  |  |  | 90.4 | 97.1 |
| Negative | 168 | 9 | 177 |  |  |
| Positive | 10 | 66 | 76 |  |  |
| Total | 173 | 73 | 246 |  |  |

**^∞^** *Asymptomatic defined as no symptoms, paucisymptomatic defined as 1–2 symptoms without anosmia or ageusia) and symptomatic defined as anosmia or ageusia, or at least three symptoms among fever; chills; severe tiredness; sore throat; cough; shortness of breath; headache; or nausea, vomiting, or diarrhoea.*

## Nationality categories

The below listed nationality categories were used in the study.

Table S4: Nationality categories

| **Nationality Category** | **Nationality** |
| --- | --- |
| **Qatar** | QATAR |
| **Northern Africa** | ALGERIA |
|  | EGYPT |
|  | LIBYA |
|  | MOROCCO |
|  | SUDAN |
|  | TUNISIA |
| **South-eastern Asia** | INDONESIA |
|  | MALAYSIA |
|  | PHILIPPINES |
|  | VIETNAM |
| **Southern Asia** | BANGLADESH |
|  | INDIA |
|  | IRAN |
|  | NEPAL |
|  | PAKISTAN |
|  | SRILANKA |
| **Western Asia** | ARMENIA |
|  | BAHRAIN |
|  | CYPRUS |
|  | IRAQ |
|  | JORDAN |
|  | KUWAIT |
|  | LEBANON |
|  | PALESTINE |
|  | REPUBLIC OF YEMEN |
|  | SAUDI ARABIA |
|  | SULTANATE OF OMAN |
|  | SYRIA |
|  | TURKEY |
|  | UNITED ARAB EMIRATES |
| **Northern America** | CANADA |
|  | USA |
| **Europe** |  |
|  | ALBANIA |
|  | BELGIUM |
|  | CROATIA (HRVATSKA) |
|  | DENMARK |
|  | FINLAND |
|  | FRANCE |
|  | GERMANY |
|  | GREECE |
|  | IRELAND |
|  | ITALY |
|  | NETHERLANDS |
|  | POLAND |
|  | PORTUGAL |
|  | ROMANIA |
|  | RUSSIAN FEDERATION |
|  | SERBIA |
|  | SPAIN |
|  | SWEDEN |
|  | UKRAINE |
|  | UNITED KINGDOM |
| **Others** | AUSTRALIA |
|  | BELIZE |
|  | BRAZIL |
|  | CENTRAL AFRICAN REPUBLIC |
|  | COLOMBIA |
|  | COMOROS |
|  | ERITREA |
|  | ETHIOPIA |
|  | JAPAN |
|  | KENYA |
|  | MAURITANIA |
|  | NICARAGUA |
|  | NIGERIA |
|  | PARAGUAY |
|  | PEOPLES REPUBLIC OF CHINA |
|  | PERU |
|  | REP. OF KAZAKHSTAN |
|  | SOMALIA |
|  | SOUTH AFRICA |
|  | SOUTH KOREA |
|  | SOUTH WEST AFRICA |
|  | TANZANIA |
|  | UZBEKISTAN |
|  | VENEZUELA |

## Prevalence ratios by age gender, nationality and municipality

Table S5: Point prevalence ratios of SARS-CoV2 by age, gender, nationality and municipality

|  | **Point Prevalence ratio (PR)** | **95% CI for PR** | **P** |
| --- | --- | --- | --- |
| **Age group (years)** | | | |
| 10-17 | Reference category |  | 0.46[NS] |
| 18-39 | 1.32 | (0.49 - 3.55) |  |
| 40-59 | 1.07 | (0.37 - 3.11) |  |
| ≥ 60 | ** | ** |  |
| **Gender** | | | |
| Female | Reference category |  | 0.79[NS] |
| Male | 1.1 | (0.55 - 2.2) |  |
| **Nationality** | | | |
| Qatar | Reference category |  | 0.11[NS] |
| Northern Africa | 1.18 | (0.24 - 5.81) |  |
| South-eastern Asia | 2.35 | (0.4 - 13.92) |  |
| Southern Asia | 4.81 | (1.39 - 16.63) ^$^ |  |
| Western Asia | 3 | (0.78 - 11.52) |  |
| Northern America | 5.02 | (0.54 - 46.91) |  |
| Europe | 1.85 | (0.19 - 17.58) |  |
| Others | 3.03 | (0.32 - 28.62) |  |
| **Municipality** | | | |
| Ad-Dawhah Municipality | Reference category |  | 0.14[NS] |
| Al Rayyan Municipality | 1.59 | (0.71 - 3.56) |  |
| Al Daayen Municipality | ** | ** |  |
| Umm Salal Municipality | 1.15 | (0.25 - 5.19) |  |
| Al Khor Municipality | 4.66 | (1.33 - 16.38)$ |  |
| Al Shamal Municipality | ** | ** |  |
| Al-Shahaniya Municipality | ** | ** |  |
| Al Wakrah Municipality | 0.34 | (0.04 - 2.64) |  |

^$^ Statistically significant at 0.05 level of significance (the 95% confidence interval for RR does not contain the null value of 1).

** Can not be calculated because one of the cells involved in cross-tabulation contains a zero

Table S6: Period prevalence ratios of SARS-CoV2 by age, gender, nationality and municipality

|  | **Period Prevalence ratio (PR)** | **95% CI for PR** | **P** |
| --- | --- | --- | --- |
| **Age group (years)** | | | |
| 10-17 | Reference category |  | 0.009 |
| 18-39 | 1.48 | (1.04 - 2.11) |  |
| 40-59 | 1.7 | (1.18 - 2.44)**^$^** |  |
| ≥ 60 | 2.04 | (1.27 - 3.28) **^$^** |  |
|  | | | |
| Female | Reference category |  | 0.028 |
| Male | 1.28 | (1.03 - 1.59) **^$^** |  |
| **Nationality** | | | |
| Qatar | Reference category |  | <0.001 |
| Northern Africa | 2.72 | (1.86 - 3.98) **^$^** |  |
| South-eastern Asia | 1.96 | (1.16 - 3.32) **^$^** |  |
| Southern Asia | 3.13 | (2.17 - 4.51) **^$^** |  |
| Western Asia | 1.76 | (1.16 - 2.68) **^$^** |  |
| Northern America | 2.2 | (0.92 - 5.24) |  |
| Europe | 0.8 | (0.32 - 1.99) |  |
| Others | 1.32 | (0.54 - 3.23) |  |
| **Municipality** | | | |
| Ad-Dawhah Municipality | Reference category |  | 0.015 |
| Al Rayyan Municipality | 0.86 | (0.66 - 1.11) |  |
| Al Daayen Municipality | 0.45 | (0.23 - 0.9) |  |
| Umm Salal Municipality | 0.9 | (0.57 - 1.43) |  |
| Al Khor Municipality | 1.36 | (0.76 - 2.42) |  |
| Al Shamal Municipality | 2.61 | (0.88 - 7.74) |  |
| Al-Shahaniya Municipality | 2.31 | (1.19 - 4.49) **^$^** |  |
| Al Wakrah Municipality | 0.86 | (0.59 - 1.26) |  |

^$^ Statistically significant at 0.05 level of significance (the 95% confidence interval for RR does not contain the null value of 1).

## Prevalence ratio by sociodemographic, lifestyle and clinical characteristics

Table S7: Period prevalence ratios of SARS-CoV2 by sociodemographic, lifestyle and clinical characteristics

|  | **Period Prevalence ratio (PR)** | **95% CI for PR** | **P** |
| --- | --- | --- | --- |
| **Highest education level** | | | |
| Never attended school | Reference category |  | 0.35[NS] |
| School | 0.85 | (0.3 - 2.44) |  |
| Technical or trade qualification | 1.3 | (0.42 - 4.01) |  |
| University | 0.95 | (0.33 - 2.71) |  |
| **Employment status** | | | |
| Employed | Reference category |  | 0.001 |
| Unemployed | 1 | (0.75 - 1.34) |  |
| Student | 0.56 | (0.4 - 0.78) **^$^** |  |
| Retired | 0.41 | (0.14 - 1.23) |  |
| **Household crowding index ^¥^** | | | |
| ≤ 1 | Reference category |  | 0.03 |
| 1.1-2 | 1.08 | (0.86 - 1.36) |  |
| >2 | 1.54 | (1.12 - 2.12) **^$^** |  |
| **BMI (kg/m^2^)** | | | |
| Healthy (18.5 – 24.9) | Reference category |  | 0.04 |
| Underweight (< 18.5) | 0.3 | (0.04 - 2.08) |  |
| Overweight (25 to 29.9) | 1.36 | (1.04 - 1.78)**^$^** |  |
| Obese (≥ 30.0) | 1.28 | (0.97 - 1.69) |  |
| **Smoking status** | | | |
| Non-smoker | Reference category |  | 0.33[NS] |
| Ex- smoker | 1.18 | (0.81 - 1.73) |  |
| Current smoker | 0.83 | (0.6 - 1.16) |  |
| **Current smoking index** |  |  |  |
| First (lowest) trecile | Reference category |  | 0.03 |
| Second trecile | 0.4 | (0.18 - 0.9) **^$^** |  |
| Third tercile (highest) | 0.5 | (0.24 - 1.05) |  |
| **Physical activity** | | | |
| 0 minutes/week | Reference category |  | 0.57[NS] |
| 1-150 minutes/week | 1.2 | (0.92 - 1.56) |  |
| 150 – 300 minutes/week | 1.01 | (0.75 - 1.36) |  |
| >300 minutes/week | 1.06 | (0.77 - 1.46) |  |
| **Fruit consumption** | | | |
| Less than once a week | Reference category |  | 0.04 |
| 2-7 times a week | 1.53 | (0.86 - 2.71) |  |
| Once or more than once a day | 1.82 | (1.04 - 3.17) **^$^** |  |
| **Vegetable or salad consumption** | | | |
| Less than once a week | Reference category |  | 0.03 |
| 2-7 times a week | 2.68 | (1.21 - 5.95) **^$^** |  |
| Once or more than once a day | 2.63 | (1.2 - 5.78) **^$^** |  |
| **Fruit juice consumption** | | | |
| Less than once a week | Reference category |  | 0.14[NS] |
| 2-7 times a week | 0.94 | (0.74 - 1.19) |  |
| Once or more than once a day | 0.82 | (0.61 - 1.11) |  |
| **BGC vaccination** | | | |
| No | Reference category |  | 0.88[NS] |
| Yes | 0.97 | (0.62 - 1.51) |  |
| **Seasonal influenza vaccination in previous 12 months** | | | |
| Yes | Reference category |  | 0.48[NS] |
| No | 1.1 | (0.85 - 1.42) |  |
| **History of chronic conditions** | | | |
| 0 | Reference category |  | 0.1[NS] |
| 1 | 1.21 | (0.93 - 1.57) |  |
| 2 | 1.16 | (0.77 - 1.75) |  |
| 3 or more | 1.79 | (1.08 - 2.97) **^$^** |  |
| **COVID-19 symptoms since start of pandemic ^∞^** | | | |
| Asymptomatic | Reference category |  | <0.001 |
| Paucisymptomatic | 1.98 | (1.51 - 2.6) **^$^** |  |
| Symptomatic | 3.51 | (2.73 - 4.51) **^$^** |  |
| **Previous close contact with an individual suspected or confirmed COVID-19** | | | |
| No | Reference category |  | <0.001 |
| Yes | 2.91 | (2.3 - 3.68) **^$^** |  |

^$^ Statistically significant at 0.05 level of significance (the 95% confidence interval for RR does not contain the null value of 1).
